# Soluble immune checkpoints in endometrial cancer – a discovery study

**DOI:** 10.3389/fimmu.2025.1721822

**Published:** 2025-11-21

**Authors:** Boštjan Pirš, Maja Novak Pušić, Luka Roškar, Tea Lanišnik Rižner, Špela Smrkolj

**Affiliations:** 1 Department of Gynaecology, Division of Gynaecology and Obstetrics University Medical Centre Ljubljana, Ljubljana, Slovenia; 2 Faculty of Medicine, University of Ljubljana, Ljubljana, Slovenia; 3 Laboratory for Translational Molecular Endocrinology, Institute of Biochemistry and Molecular Genetics, Faculty of Medicine, University of Ljubljana, Ljubljana, Slovenia; 4 Department of Gynaecology and Obstetrics, General Hospital Murska Sobota, Murska Sobota, Slovenia

**Keywords:** endometrial cancer, prognostic biomarker, predictive biomarker, diagnosticbiomarker, soluble immune checkpoint, immune checkpoint inhibitor, lymphovascular spaceinvasion, mismatch repair deficiency

## Abstract

**Background:**

Soluble immune checkpoints (sICs) are circulating forms of membrane-bound immune molecules, the latter being targets of immune checkpoint inhibitors, widely used in cancer treatment. Altered sIC levels have been reported in several malignancies and sICs are posited as promising diagnostic, prognostic and predictive biomarkers measurable in peripheral blood. However, data on their levels in endometrial cancer (EC) patients are scarce. This study aimed to evaluate plasma concentrations of multiple sICs in EC patients and assess their potential diagnostic, prognostic, and predictive value.

**Methods:**

In this prospective case–control study, plasma levels of 16 soluble immune checkpoints were measured in 50 patients with histologically confirmed EC prior to surgical staging and in 26 age- and BMI-matched controls undergoing benign gynecologic surgery. Fluorescence-based multiplex immunoassay (MagPix, Luminex) was used to quantify analyte concentrations. FIGO 2023 stage classification and risk grouping according to ESGO 2020 guidelines was performed based on clinicopathologic data and molecular characteristics (MMR and p53 status). Statistical analyses were performed using non-parametric tests and robust logistic regression.

**Results:**

EC and control groups did not differ in demographic, clinical, or lifestyle parameters. sIC levels were measurable in majority of patients. No significant differences in sIC levels were observed between EC patients and controls. Within the EC cohort, patients with MMR-deficient tumors exhibited significantly elevated levels of sPD-1, sPD-L1, sLAG-3, sICOS, sGITR, and sCD86 compared with MMR-proficient cases. Higher plasma concentrations of sTIM-3, sCD27, sHVEM, and sCD40 were associated with the presence of lymphovascular space invasion (LVSI). Levels of sCD27 and sCD40 were significantly higher in advanced or metastatic disease (stage IIIA or higher).

**Conclusions:**

Although soluble immune checkpoint levels did not differentiate EC patients from controls, several sICs correlated with key prognostic and predictive features, including LVSI, advanced stage, and MMR deficiency. These findings suggest that circulating immune checkpoint proteins may serve as non-invasive biomarkers for risk assessment and immunotherapy response prediction in endometrial cancer. Further validation in larger, independent cohorts is warranted.

## 1 Introduction

Endometrial cancer (EC) is the fourth most common cancer in women and the most common gynecological cancer in the developed world, the incidence and prevalence continue to rise (1). In majority of cases, the diagnosis of endometrial cancer is straightforward, made by histopathological examination of tissue obtained by an invasive procedure – hysteroscopy, endometrial biopsy or uterine curettage. However, decision for invasive intervention in clinical and ultrasound borderline cases presents a dilemma. In practice, a large number of invasive procedures are performed to find a minority of patients ultimately diagnosed with endometrial cancer (2, 3). A non-invasive test based on peripheral blood biomarkers would facilitate triage of patients to observation or invasive diagnostic intervention.

In addition to information gained from diagnostic intervention (histological type, molecular classification) and imaging, information obtained with surgical intervention – hysterectomy and staging (definitive anatomical stage, especially lymph node status and presence of lymphovascular space invasion) are required for the final staging of the tumor according to FIGO 2023 and risk group stratification which determine the need for adjuvant therapy (4, 5). The detection of biomarkers from blood plasma that are associated with prognosis could have a significant impact on routine clinical practice, especially in patients with a wish for fertility preservation which are not undergoing surgical therapy.

While patients diagnosed at an early stage exhibit excellent survival, the prognosis of advanced or metastatic disease is dismal, with a 5-year overall survival of only 17%. Until recently, therapy for relapsed disease or metastatic disease have been limited to platinum-based chemotherapy or hormonal therapy conferring a modest median overall survival of only 12 months (5, 6). Immune checkpoint inhibitor (ICI) therapy represents new standard for specific subgroups of these patients, most notably those with mismatch repair (MMR) deficient tumors (7). Biomarkers that predict response to ICI therapy (e.g. presence of tumor infiltrating lymphocytes, PD-L1 expression, tumor mutational burden, MMR deficiency) are determined by immunohistochemistry (IHC) or genetic methods and require tumor tissue. This approach may be limited by the spatial and temporal biomarker heterogeneity of tumors. Namely, the tumor sample obtained by biopsy may not represent entire tumor cell population. Additionally, biomarker expression, biological behaviour and response to ICI therapy may change during the treatment or progression – the sample obtained at diagnosis may not be representative of the tumor biomarker status at the time that ICI therapy is considered. Repeat biopsies mean additional invasive interventions and potentially delay the start of treatment (8–11). The discovery of novel peripheral blood biomarkers associated with response to ICI therapy in endometrial cancer is potentially transferable to routine clinical use after multicenter validation. Biology of anticancer immune response is uniform across various cancers, therefore the discovery of novel immunotherapy response predictive biomarkers could be transferable to other malignancies, following further research.

Targets of immune checkpoint inhibitors are proteins that are located on the membranes of immune cells as well as tumors and other cells. Due to the phenomenon of alternative intron splicing or cleavage by proteolytic enzymes, soluble forms are produced simultaneously with transmembrane counterparts, and their levels can be measured in plasma or serum from peripheral blood. Initially soluble immune checkpoints (sICs) were perceived only as a by-product of the synthesis of transmembrane forms, but more recently they have also described their biological role in terms of systemic or paracrine control of the immune system, similar to cytokines. Several studies suggest that these molecules could be used for non-invasive diagnostics, prognosis, and prediction of treatment response in lung, gastric, renal, urothelial, pancreatic, colorectal, head and neck and other cancer patients (9, 12–15).

Based on previous extensive research on transmembrane counterparts can soluble immune checkpoints be simplistically classified as inhibitory, stimulatory and of mixed roles. The most well researched soluble immune checkpoints are inhibitory CTLA-4/CD28: CD80(B7-1)/CD86(B7-2) and PD-1:PD-L1/PD-L2. Less extensively studied inhibitory and stimulatory checkpoints include TIM-3:GAL-9, LAG3:MHC, BTLA: HVEM and ICOS: ICOSL, OX40:OX40L, GITR: GITRL, CD40:CD40L, CD27:CD70, respectively (9, 12–14).

Aim of the presented study was to discover potential peripheral blood diagnostic, prognostic and predictive biomarkers in endometrial cancer among a larger set of soluble immune checkpoints.

## 2 Materials and methods

### 2.1 Study design and participants

This was a prospective case–control study conducted at the Department of Gynecology, University Medical Centre (UMC) Ljubljana, Slovenia. The study included 50 patients with histologically confirmed endometrial cancer (EC) and 26 control patients without significant comorbidities undergoing surgery for benign gynecological conditions, predominantly uterovaginal prolapse, urinary incontinence, or uterine leiomyomas. Patients were enrolled between November 2018 and October 2020. Patients from both groups were enrolled before surgery. Control participants were matched to EC patients based on age and body mass index (BMI) to minimize confounding effects.

All participants received detailed verbal and written information about the study and provided signed informed consent prior to enrollment. The study protocol was reviewed and approved by the National Medical Ethics Committee of Slovenia (approval no. 0120-515-2017/4).

### 2.2 Inclusion and exclusion criteria

Eligible EC patients were required to have a confirmed preoperative diagnosis of endometrial carcinoma before undergoing surgical staging or other oncologic treatment. The following exclusion criteria were applied to all participants: 1) presence of malignant neoplasm of other sites– before or during primary treatment or within 2 years of primary treatment, 2) history of recurrent malignant neoplasm of other sites, 3) current or past recurrent endometrial cancer, 4) presence of atypical endometrial hyperplasia (applicable only to control group), 5) therapy with immunosuppressive or immunomodulatory medications excluding inhalatory or topical corticosteroids, 6) pregnancy, 7) age less than 18 years.

### 2.3 Diagnostic and histopathological evaluation

All EC patients underwent preoperative imaging according to the institutional protocol. This included expert transvaginal ultrasound assessment by a gynecologic oncologist and abdominal ultrasound for localized, non-aggressive tumors. Patients with aggressive histotypes or suspected advanced disease underwent abdominal computed tomography (CT) for staging purposes.

Surgical specimens were examined by dedicated gynecologic pathologists and reported according to the International Collaboration on Cancer Reporting (ICCR) dataset. Immunohistochemical (IHC) analysis was performed for p53 to determine aberrant versus wild-type expression, and for mismatch repair (MMR) proteins (MLH1, PMS2, MSH2, and MSH6) to determine MMR status – deficient or proficient. Lymph node evaluation was performed histologically using hematoxylin–eosin (HE) staining for patients who underwent systematic lymphadenectomy and by ultrastaging for those who underwent sentinel lymph node biopsy (16). Lymphovascular space invasion pattern was stratified according to ESGO 2020 guidelines into absent/focal and substantial, the latter category being defined by multifocal or diffuse arrangement of LVSI or the presence of tumor cells in five or more lymphovascular spaces (5). Tumors were stratified into non-aggressive (grade 1 and 2 endometrioid) and aggressive (all others) in line with FIGO 2023 staging. EC patients were stratified to stages and risk groups according to FIGO 2023 staging and ESGO 2020 guidelines, respectively (4, 5).

### 2.4 Clinical and lifestyle data collection

Comprehensive demographic and clinical data were obtained for all participants, including personal and family medical history, gynecologic and obstetric history, and current medication use. Obesity was defined as a BMI ≥30 kg/m², in accordance with World Health Organization (WHO) criteria.

Lifestyle information was collected using a structured questionnaire, covering average weekly physical activity volume, alcohol consumption, and smoking status.

### 2.5 sIC levels measurement

During routine preoperative blood work, additional peripheral venous blood study samples were collected. Patients were fasted for food (>8 hours since last meal) and allowed non-carbohydrate drinks (e.g. water, coffee without sugar) before sample collection. Strict and detailed standard operating procedures were followed in sample collection and processing. Specifically, 6mL of peripheral venous blood was collected into BD vacutainer K2 EDTA tubes (Cat. No#: 367864, BD Medical, New Jersey, USA). To assure complete mixing of blood with EDTA were the tubes inverted 10 times immediately after venipuncture. Collected blood was centrifuged within 1 hour of collection at 1400g for 10 minutes at 4°C. Obtained plasma was transferred to a 5mL polypropylene tube (Cat. No#:352063, BD Medical), mixed several times with a disposable plastic Pasteur pipette during transfer. Plasma samples were divided into 200 μl aliquots and stored in cryogenic tubes (Cat. No#: 375418, Thermo Scientific, Waltham, Massachusetts, USA) at– 80°C until further analysis.

**Figure 1:**
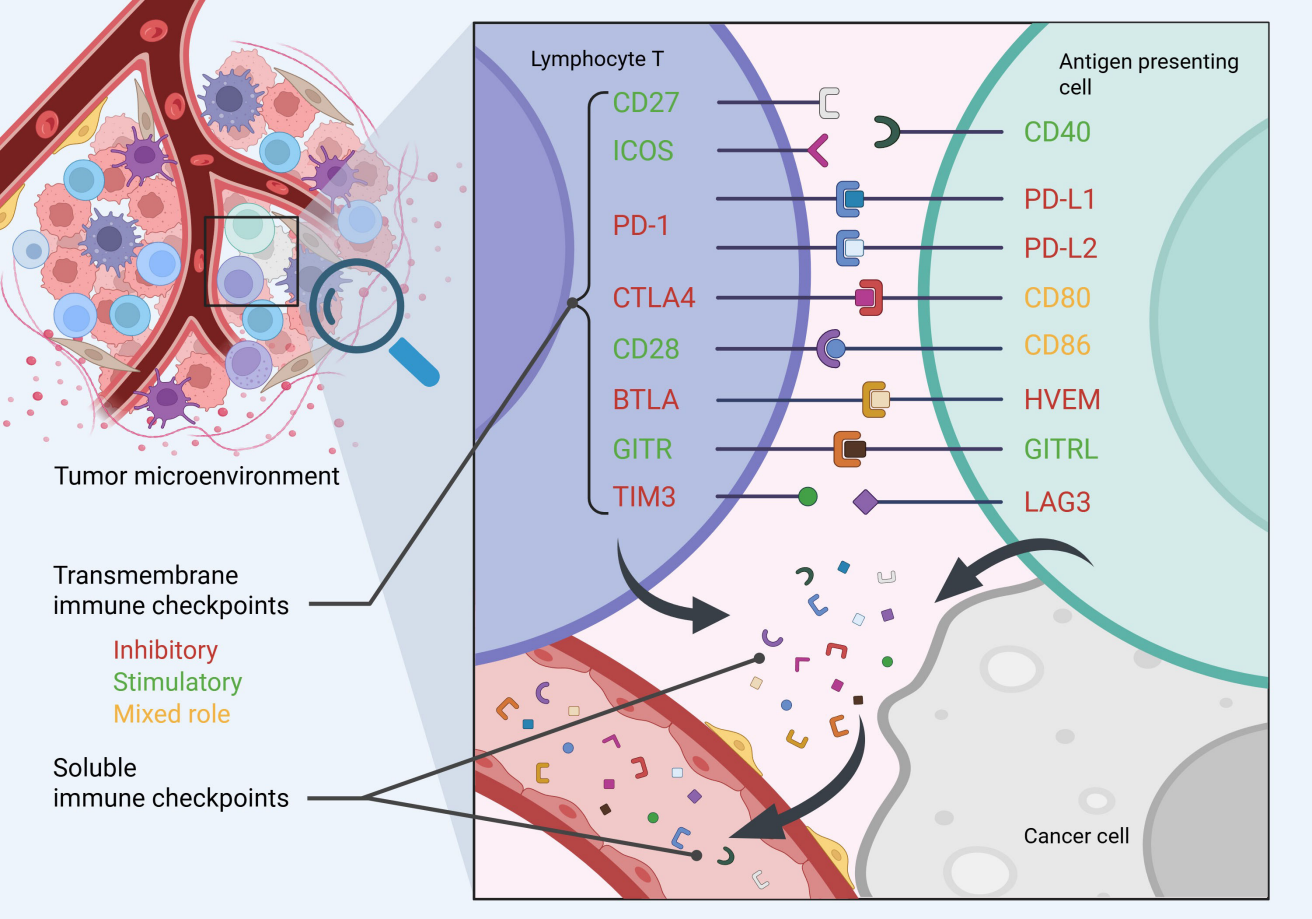
Schematic representation of soluble immune checkpoints (sICs), measured in the study.

Levels of 16 different soluble immune checkpoints were measured – 8 with predominantly inhibitory role (sPD-1, sPD-L1, sPD-L2, sCTLA-4, sLAG3, sTIM3, sBTLA, sHVEM), 6 with a predominantly stimulating role (sICOS, sGITR, sGITR-L, sCD40, sCD27, sCD2) and 2 with dual role (sCD80, sCD86) (**Figure 1**). We utilized multiplex sandwich immunosorbent assay – Human Immuno-Oncology Checkpoint Protein Panel 1 - HCPK1-11K-PX17 (Lot No: #: 3905113, Merck Millipore, Burlington, Massachusetts, USA), on MagPix platform (Luminex, Austin, Texas, USA). Assay was performed according to protocol supplied by the manufacturer. According to manufacturer’s specifications, intra-assay and inter-assay coefficient of variation does not exceed 5% and 10%, respectively, for all measured analytes. All sIC levels for all included patients were measured in one run, thereby limiting the issue with inter-assay variability.

Fluorescence intensities obtained from the MagPix were subtracted by background fluorescence. Standard samples provided with the assay were used to generate calibration curves used to determine analyte concentrations in study samples. The results were expressed in picograms per milliliter (pg/mL). Data acquisition and initial processing were performed using xPonent software (Luminex, Austin, TX, USA) and Bio-Plex Manager software (Bio-Rad Laboratories, Hercules, CA, USA).

### 2.6 Data analysis and statistical methods

Patients’ and tumor characteristics were stored anonymously in computer database. All further statistical analysis was done using jamovi and JASP, graphical user interfaces for R statistical programming language (17, 18).

Due to violation of assumptions of general linear model and non-normal distributions of variables, we used median as a measure of central tendency and non-parametric or robust versions of statistical tests. Specifically, histopathologic, clinical and lifestyle characteristics were described by median and interquartile range (IQR) or absolute and relative frequencies. Between-group comparison was performed with Mann Whitney U test or Kruskar-Wallis test for numerical variables and Chi-square test for categorical variables. sIC levels among subgroups were compared with Kruskar-Wallis or Mann-Whitney U test. Robust logistic regression was used to compare sIC levels between EC patients and controls, controlled for potential cofounders that were preselected among previously validated endometrial cancer risk factors, namely age, body mass index, waist-to-hip ratio, physical activity (average number of hours of physical activity per week), parity, menopausal status, smoking status. No multiple comparison correction was applied and reported p-values are nominal (19).

## 3 Results

### 3.1 Clinical and tumor characteristics

EC patients did not significantly differ to control patients in respect to age, BMI, waist-to-hip ratio, presence of obesity, menopausal status, age at menopause, history of infertility, parity, average number of hours of physical activity per week, alcohol consumption and smoking status (**Table 1**). Likewise, there were no significant differences between groups regarding comorbidities or current medication use (**Supplementary Table 1**).

**Table 1:** Clinical and lifestyle characteristics of study patients expressed in median (interquartile range) or absolute (relative) frequencies.

Clinical and lifestyle characteristics				
	All patients (N = 76)	Control patients (N = 26)	EC patients (N = 50)	EC vs control patients, p-value
Age, median (IQR)	64.0 (55.0, 70.0)	63.5 (55.2, 68.0)	64.0 (55.2, 70.0)	0.771 (1)
BMI (kg/m^2^), median (IQR)	29.1 (25.4, 33.1)	29.8 (26.8, 33.0)	28.9 (25.0, 33.0)	0.661 (1)
Waist to hip ratio, median (IQR)	0.860 (0.810, 0.920)	0.865 (0.795, 0.915)	0.860 (0.825, 0.915)	0.608 (1)
Obesity	N (%)	N (%)	N (%)	0.740 (2)
No	40 (52.6%)	13 (50.0%)	27 (54.0%)	
Yes	36 (47.4%)	13 (50.0%)	32 (46.0%)	
Menopausal status	N (%)	N (%)	N (%)	0.220 (2)
Postmenopausal	62 (81.6%)	19 (73.1%)	43 (86.0%)	
Premenopausal	14 (18.4%)	7 (26.9%)	7 (14.0%)	
Age at menopause, median (IQR)	52.0 (50.0, 53.0)	52.0 (50.0, 53.0)	52.0 (50.0, 53.0)	0.880 (1)
History of infertility	N (%)	N (%)	n (%)	1.00 (2)
No	69 (94.5%)	25 (96.2%)	44 (93.6%)	
Yes	4 (5.5%)	1 (3.8%)	3 (6.4%)	
Parity	N (%)	N (%)	N (%)	0.150 (2)
0	3 (4.1%)	1 (3.8%)	2 (4.3%)	
1	15 (20.5%)	2 (7.7%)	13 (27.7%)	
2	41 (56.2%)	16 (61.5%)	25 (53.2%)	
>2	14 (19.2%)	7 (26.9%)	7 (14.9%)	
Physical activity (average hours/week), median (IQR)	27.8 (20.0, 35.2)	27.0 (19.3, 33.1)	27.8 (22.1, 35.8)	0.960 (1)
Alcohol consumption	N (%)	N (%)	N (%)	1.00 (2)
No	58 (80.6%)	21 (80.8%)	37 (80.4%)	
Yes	14 (19.4%)	5 (19.2%)	9 (19.6%)	
Smoking status	N (%)	N (%)	N (%)	1.00 (2)
Non-smoker	62 (86.1%)	22 (84.6%)	40 (87.0%)	
Smoker	10 (13.9%)	4 (15.4%)	6 (13.0%)	

BMI, body mass index; N, number (absolute frequency), % - percent (relative frequency). IQR, interquartile range. (1) – Mann Whitney U test. (2) – Chi square test.

There were no missing values. No significant differences between endometrial cancer (EC) patients and control patients in numerical (Mann-Whitney U test) or categorical variables (Chi square test) were observed. Obesity defined as BMI > 30 kg/m^2^ as per WHO.

Among EC patients, 66% had tumors of non-aggressive histological subtypes (grade 1 or 2 endometrioid EC). MMR deficiency was identified in 33% of tumors, while 14% exhibited aberrant p53 expression. Most patients had tumors that were confined to the uterus, whereas 16% of patients presented with stage IIIA disease or higher. Lymphovascular space invasion (LVSI) was present in 32% of cases. Lymph node staging was performed in 45 (90%) EC patients, among whom 5 (11%) had positive nodes (**Table 2**).

**Table 2:** Tumour characteristics in endometrial cancer patients.

	EC patients (N = 50)	
Histological type, n(%)	Endometrioid	41 (82.0%)
	Serous	6 (12.0%)
	Mixed	2 (4.0%)
	Other	1 (2.0%)
Endometrioid cancer grade, n(%)	1	20 (47.6%)
	2	13 (31.0%)
	3	9 (21.4%)
Histological aggressiveness (FIGO 2023), n(%) ^a^	Non-aggressive	33 (66.0%)
	Aggressive	17 (34.0%)
LVSI, n (%) ^b^	Absent/focal	34 (68.0%)
	Substantial	16 (32.0%)
Lymph node staging performed, n(%)	Yes	45 (90.0%)
	No	5 (10%)
Positive lymph nodes, n(%)	No	40 (88.9%)
	Yes	5 (11.1%)
MMR status, n(%) ^c^	Deficient	16 (32.0%)
	Proficient	34 (68.0%)
p53 status, n(%) ^d^	Abberant	7 (14.0%)
	Wild type	43 (86.0%)
Stage (FIGO 2023), n(%)	IA1	11 (22.0%)
	IA2	7 (14.0%)
	IA3	0 (0.0%)
	IB	2 (4.0%)
	IC	6 (12.0%)
	IIA	3 (6.0%)
	IIB	8 (16.0%)
	IIC	3 (6.0%)
	IIC(IICmp53ab)	2 (4.0%)
	IIIA	0 (0.0%)
	IIIB	0 (0.0%)
	IIIC1	3 (6.0%)
	IIIC2	2 (4.0%)
	IVA	0 (0.0%)
	IVB	3 (6.0%)
	IVC	0 (0.0%)
Risk group (ESGO 2020), n(%) ^e^	LR	18 (36.0%)
	IR	8 (16.0%)
	HIR	14 (28.0%)
	HR	7 (14.0%)
	AM	3 (6.0%)

LR, low risk; IR, intermediate risk; HIR, high intermediate risk; HR, high risk; AM, advanced/metastatic disease (stage III or higher with residual disease after surgery or stage IV), LVSI, lymphovascular space invasion; MMR, mismatch repair

Numbers of patients in subcategories represented by absolute (n) and relative frequency (%) ^a^Non aggressive tumors defined as endometrioid low grade (grade 1 or 2) and aggressive as all others according to FIGO 2023 staging. ^b^LVSI defined by ESGO-ESTRO-ESP 2020 guidelines criteria. ^c^Mismatch repair status was determined by expression of MLH1, PMS2, MSH2 and MSH6 by IHC – tumors with absent expression in one or more of those proteins were deemed as deficient, otherwise as proficient. ^d^Expression of p53 was determined as abberant or wild type by IHC. ^e^Risk groups according to ESGO-ESTRO-ESP 2020 guidelines

### 3.2 Soluble immune checkpoints are measurable in majority of EC and control patients

Levels of sPD-L2 and sLAG-3 levels were successfully measured in all samples, sCTLA4 levels in all but two samples and sCD86 levels in all but 5 samples. Other sIC levels were measurable in all but one sample. All samples not measured were due to being lower to minimum detectable concentration. Levels of sPD-L1, sBTLA, sGITR, sGITRL and sCD80 were extrapolated in 1 or 2 samples, whereas levels of sCTLA-4 and sCD86 were extrapolated in 29 and 10 samples, respectively. All extrapolated samples were below lower limit of quantification. Other sIC levels were within standard curve for all measurable samples. Further analysis was performed in both versions – including and excluding extrapolated values. Except to association of MMR status with sICs levels, no differences in significance of results between analysis including and excluding extrapolated values have been observed. Control patients’ inter-individual coefficient of variation in sIC levels was below 30% for sPD-L2, sTIM3, sCD40, and varied between 30% and 66% for others (**Supplementary Table 2**).

No significant differences in the levels of individual soluble immune checkpoints were observed between EC patients and control patients (**Supplementary Table 3**).

No individual sIC level was significantly associated with diagnosis of endometrial cancer after adjusting for age, body mass index, waist-to-hip ratio, physical activity, average number of hours of physical activity per week, parity, menopausal status, smoking status (**Supplementary Table 3**).

### 3.3 Plasma sIC levels were associated with MMR status, presence of LVSI, EC stage and risk

To evaluate whether soluble immune checkpoint levels were associated with established prognostic and predictive factors in EC, we compared sIC concentrations across subgroups defined by FIGO 2023 stage, ESGO 2020 risk group, histopathological type (aggressive vs. non-aggressive), MMR status, LVSI, and p53 expression pattern.

Levels of sICs did not significantly differ by the histopathological type (aggressive vs non-aggressive), and type of p53 expression (p<0.05, Mann-Whitney U test) (**Supplementary Tables 4**, **5**).

**Figure 2:**
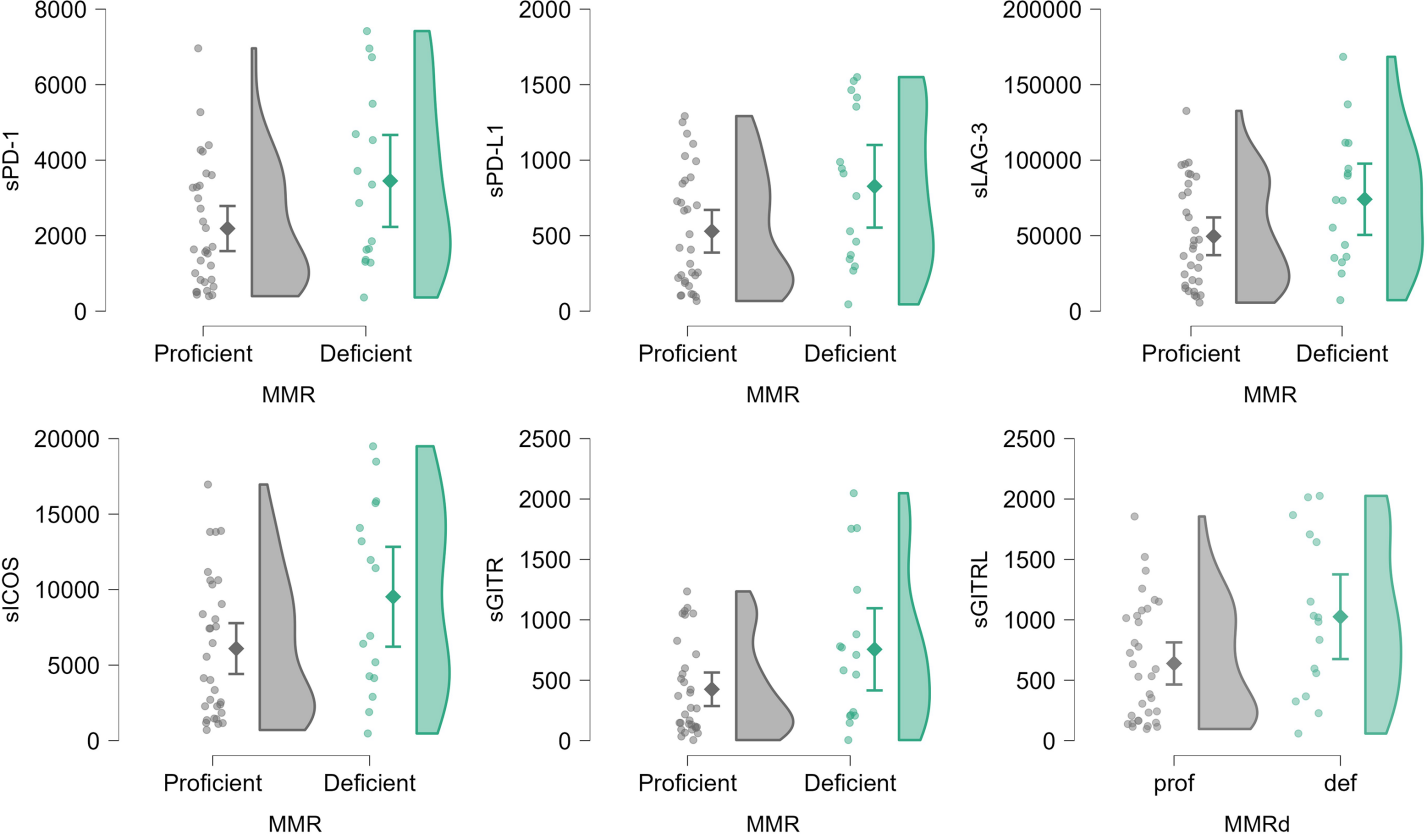
Raincloud plots of significant (for sPD-1, sPD-L1, sLAG3, sICOS, sGITR, p<0.05, Mann-Whitney U test) and marginally significant (for sGITRL, p= 0.050, Mann-Whitney U test) differences in sIC levels by tumor mismatch repair status (MMR), determined as deficient if the expression of at least one of the MMR proteins is absent and as proficient otherwise. Extrapolated values in 1 or 2 patients for sPD-L1, sGITR, sGITRL are included. Levels are expressed in pg/mL. Plotted from left to right are jittered data points, mean and 95% CI, kernel density plot.

Levels of sPD-1, sPD-L1, sLAG-3, sICOS, sGITR and sCD86 were significantly higher in mismatch repair deficient tumors (p<0.05, Mann-Whitney U test) and difference in sGITRL was marginally significant (p=0.050, Mann-Whitney U test). (**Figure 2**, **Supplementary Table 5**). In analysis without extrapolated sIC levels, significance of differences in sCD86 levels was lost, and differences in sCTLA4 levels were significant but the number of patients was much lower, due to large number of patients with extrapolated levels (p=0.014, N = 26, Mann-Whitney U test).

**Figure 3:**
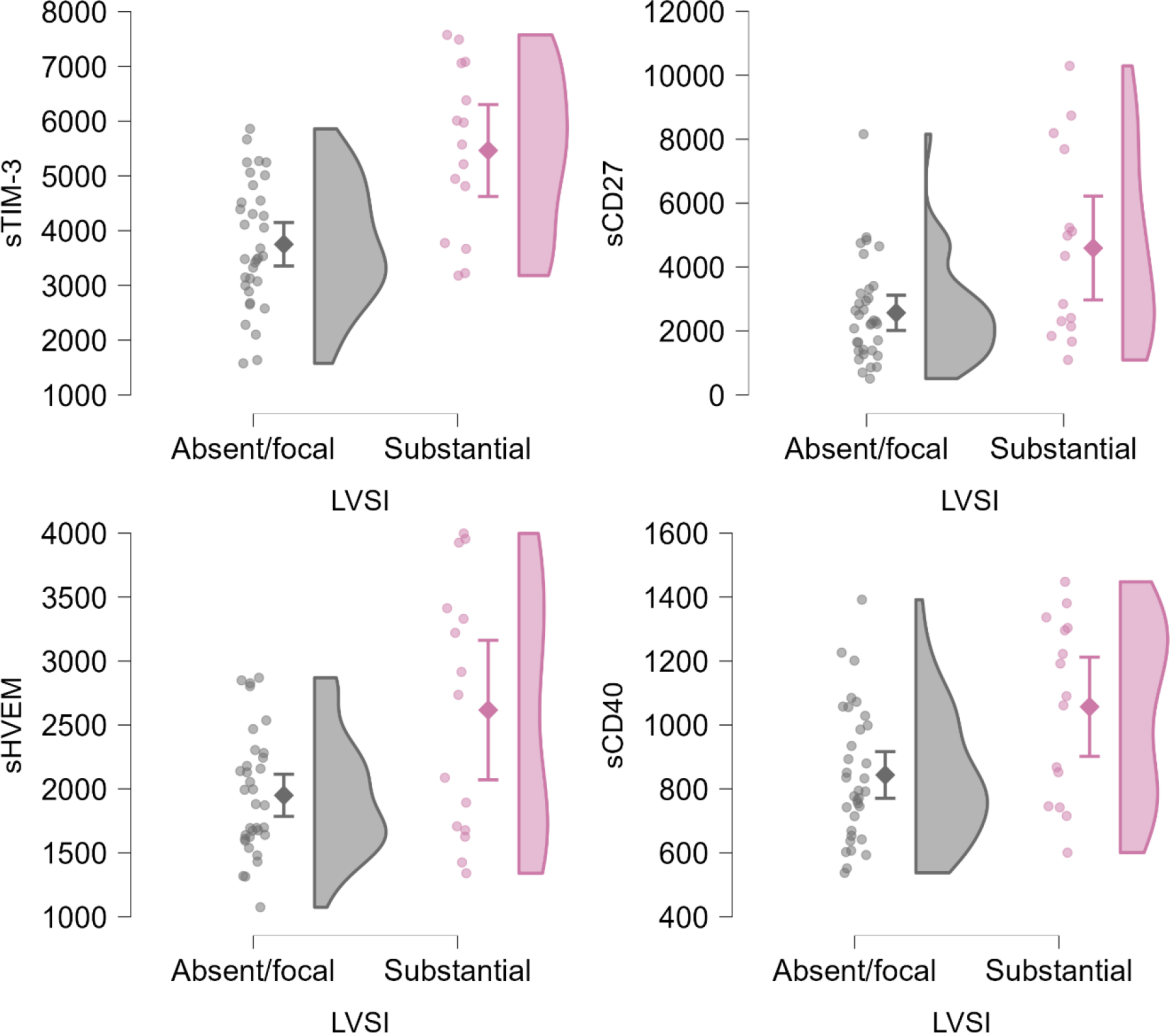
Raincloud plots of significant differences (p<0.05, Mann-Whitney U test) in sIC levels by lymphovascular space invasion (LVSI) extent defined as absent or focal and substantial as per ESGO-ESTRO-ESP 2020 guidelines criteria. Levels are expressed in pg/mL. Plotted from left to right are jittered data points, mean and 95% CI, kernel density plot.

Levels of sTIM-3, sCD27, sHVEM and sCD40 were significantly higher in patients with the tumors that had presence of lymphovascular space invasion (**Figure 3**, **Supplementary Table 4**).

**Figure 4:**
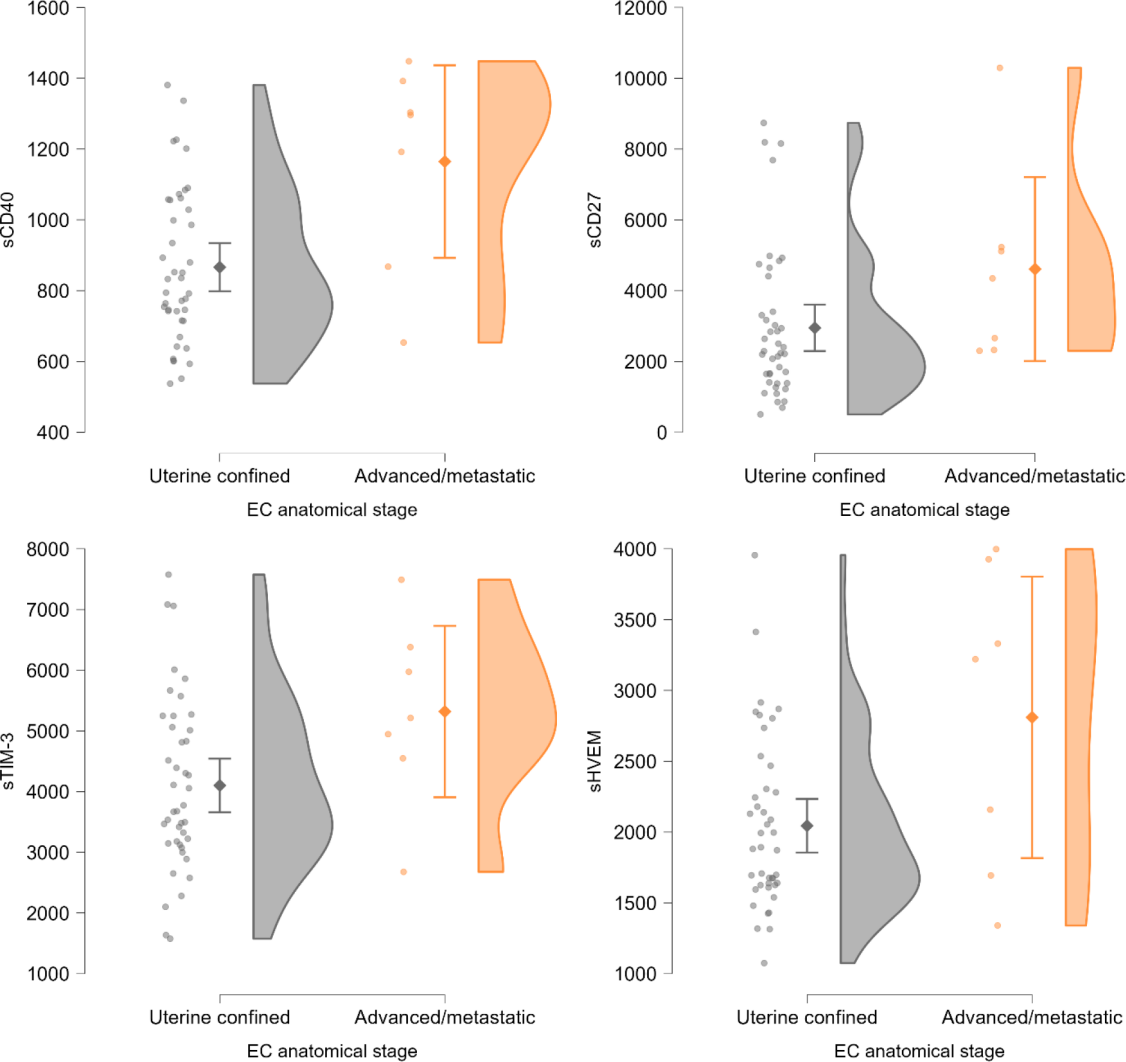
Raincloud plot of differing TIM3 and HVEM levels by ESGO 2020 guidelines risk group, significant for TIM-3 (Kruskar-Wallis test, p=0.01). Levels are expressed in pg/mL. Plotted from left to right are jittered data points, box and whiskers plot with added mean, kernel density plot. LR, low risk; IR, intermediate risk; HIR, high intermediate risk; HR, high risk; AM, advanced/metastatic disease (stage III or higher with residual disease after surgery or stage IV).

Levels of sTIM-3 and sHVEM tended to be higher in patients with higher FIGO 2023 stage tumors (sig. for sHVEM, p =0.01, Kruskar-Wallis test), and higher ESGO 2020 guidelines risk group (sig. for sTIM3, p = 0.01, Kruskar-Wallis test), mainly due to elevated levels in stage IV or advanced/metastatic disease (**Figure 4**).

**Figure 5:**
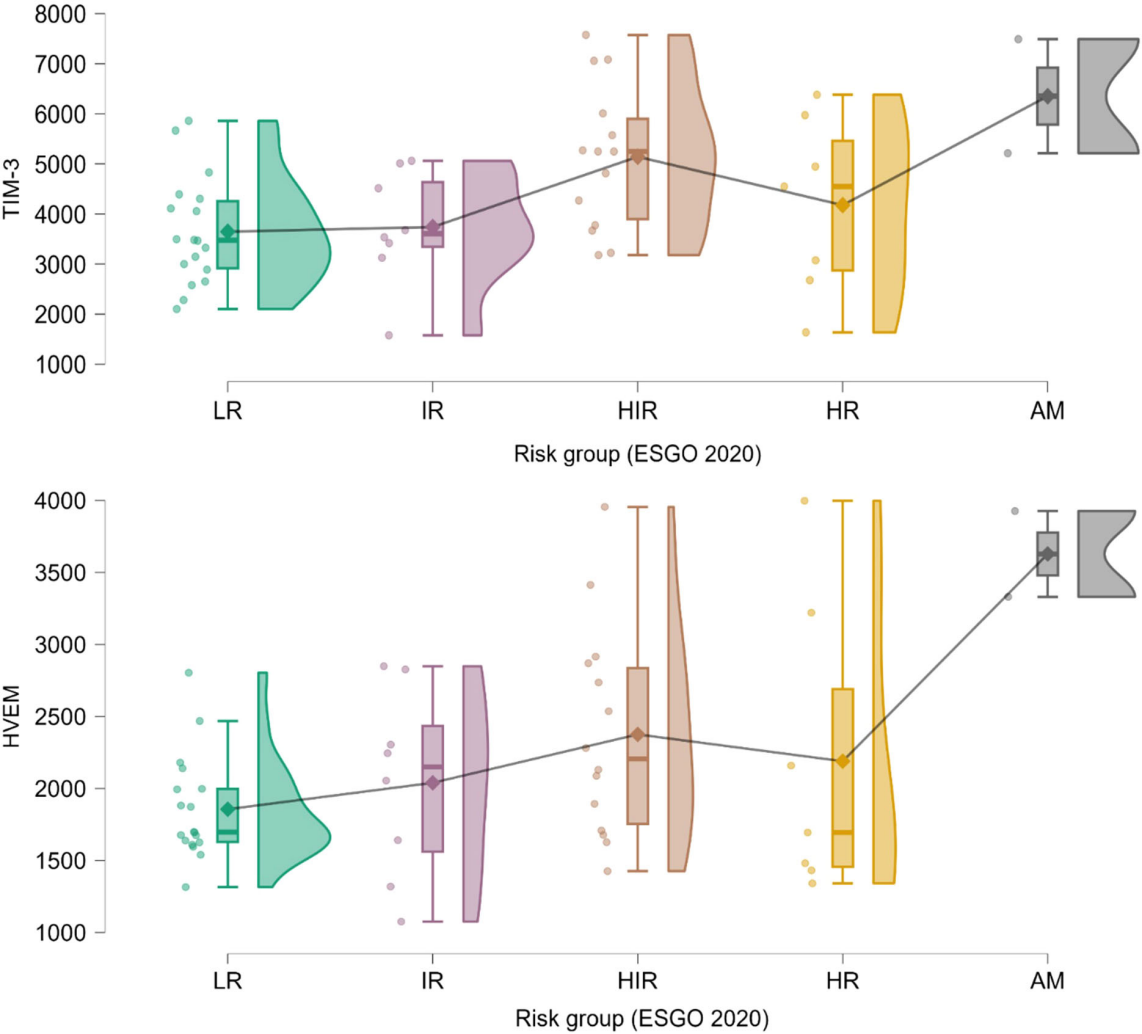
Raincloud plots of sIC levels by anatomical stage - differences between patients with uterus confined disease (FIGO 2023 stage IIC or lower) and patients with locally or regionally advanced or metastatic disease (FIGO 2023 stage IIIA or higher). Significant for sCD40 and sCD27 (p<0.05, Mann-Whitney U test). Levels are expressed in pg/mL. Plotted from left to right are jittered data points, mean and 95% CI, kernel density plot.

When comparing patients with disease confined to the uterus (stage IIC or lower) to patients with locally advanced or metastatic disease (stage IIIA or higher), sCD27 and sCD40 levels were significantly higher in patients with advanced disease (p<0.05, Mann-Whitney test) and similarly, sTIM-3 and sHVEM levels tended to be higher in the latter, although non-significantly (**Supplementary Table 6**, **Figure 5**).

## 4 Discussion

Differing levels in soluble immune checkpoints have previously been demonstrated in various malignancies suggesting these molecules as potential biomarkers for non-invasive cancer diagnosis (9, 12, 13, 20, 21). However, only one published study to date explored differing sICs levels between endometrial cancer patients and healthy controls. Authors compared 23 EC patients and 11 controls, unveiling small difference in sPD-L1 (mean 153 vs. 103 pg/mL) and marked differences in sPD-L2 levels (mean 1331 vs. 172 pg/mL) (21). In present study, we did not discover any significant or near-significant differences in any of the studied sIC levels between EC patients and controls. Included EC patients had mainly localized disease of non-aggressive histological type, similarly to the EC patients in previous study. In present study, included control patients were similar to EC patients in regards to clinical and lifestyle characteristics and therefore represented clinically relevant control sample. Contrary to patients, control patients are not well profiled in previous study, therefore differences in control patients may have caused discrepancy in observed results. In addition, in present study were the inter-individual coefficients of variation in control patients moderate or large for most of the measured sIC levels. If confirmed in further studies, this fact may preclude these analytes as robust diagnostic biomarkers in routine clinical use.

Endometrial cancer carries an overall good prognosis. With rising incidence of the disease and rising life expectancy in developed countries, quality of life of cancer survivors is getting increasingly important. As a result, there are tendencies to deescalate cancer treatment in patients deemed to have low risk disease. However, endometrial cancer is a heterogenous disease. Current risk stratification relies on a combination of clinicopathologic and molecular features, but these approaches are still imperfect, and there is a recognized need for reliable, preferably non-invasive, prognostic biomarkers (1, 22). Soluble immune checkpoints have been studied as prognostic biomarkers in a range of cancers. In addition to comparing EC patients and controls, study by Mamat et al. explored differing levels of sPD-1, sPD-L1 and sPD-L2 by some established prognostic biomarkers (histological type, grade, presence of LVSI, stage, myometrial invasion) in 23 EC patients but failed to demonstrate any differences. Conversely, study by Kontomanolis et al. which included 19 EC patients showed a significant association of elevated sPD-L1 levels with higher histological grade and locally advanced stage. However, the number of the included EC patients in previous studies was low, and, more importantly, EC patients were not characterized with molecular markers. In presented study we explored association of plasma soluble immune checkpoint levels with a range of EC prognostic features. We discovered significant and potentially clinically meaningful differences in levels of sTIM-3, sCD27, sHVEM and sCD40 in EC patients that had tumors that exhibited lymphovascular space invasion. Specifically, levels of all were higher in the presence of LVSI. This finding may be especially clinically relevant due to LVSI being an important prognostic marker in early-stage disease and its presence is often deciding factor on postoperative adjuvant therapy. Furthermore, contrary to molecular classification, anatomical stage and other histopathologic features, it cannot be determined preoperatively by biopsy or by imaging, as it can be determined only with histopathological examination of hysterectomy specimen. This fact may be especially relevant in a clinical setting of younger EC patients desiring treatment with preservation of fertility. When comparing EC patients among composite risk factors, namely FIGO 2023 stage and ESGO 2020 risk group, sTIM-3 and sHVEM levels tended to be elevated in higher stage or higher risk group tumors, showing partial dose response pattern and the biggest difference observed when comparing stage IV or advanced/metastatic group to others. Due to multiple subgroups it is possible that this analysis was underpowered to detect differences in other sIC levels. To counteract this, EC patients were divided two subgroups based on anatomical stage – to those with uterus confined disease and those with locally and regionally advanced or metastatic disease. In this analysis, levels of sCD27 and sCD40 were significantly higher and levels of sTIM-3 and sHVEM were near-significantly higher in advanced/metastatic disease.

Additionally, we observed significantly higher sPD-1, sPD-L1, sLAG-3, sICOS and sGITR levels in EC patients with mismatch repair (MMR) deficient tumors. MMR is an established biomarker in endometrial cancer, as well as other malignancies, being associated with adverse clinicopathologic features and worse survival (23). More importantly, it is one of the established immunotherapy response predictive biomarkers and only one that is in widespread use in endometrial cancer. Briefly, due to lacking DNA repair mechanism, MMR deficient tumors cells carry more mutations in their genome and are therefore more immunogenic, stimulating endogenous immune response and making these patients more responsive to checkpoint inhibition (8, 11). This may also be a theoretical basis for elevated levels of sICs in these tumors. Currently, immunotherapy is reserved for EC patients with recurrent or primary metastatic or advanced disease. Spatial and temporal heterogeneity of MMR deficiency, being a tissue biomarker, is one of the recognized limitations that could be overcome by the use peripheral blood based biomarkers such as sICs (10, 11).

Presented study is the first to examine peripheral blood concentrations of larger set of 16 soluble immune checkpoints in patients with endometrial cancer. Its strengths include: the patients included were comprehensively characterized on clinical, pharmacologic, lifestyle and tumor properties, tumors were characterized according to currently used guidelines and staging system. Tumors were molecularly characterized by MMR expression and p53 expression status. Studied cohort of EC patients was representative of a typical EC patient population in developed countries in regards to histological type, aggressiveness, stage, risk group, MMR status, presence of LVSI, positive lymph nodes status (1, 22, 24). Control and EC patients were well matched on clinical and lifestyle characteristics. Blood sampling was carried out under strict standard operating procedures and all sIC levels for all included patients were measured withing one assay run, ensuring equivalent sampling, processing and measuring conditions for both groups. Limitations are moderate size of EC patient group and small size of control patient group and the fact that the groups were not balanced in size. However, this design was intentionally chosen, as the majority of the investigated hypotheses involved subgroups of EC patients. SCTLA4 sCD86 levels were extrapolated for significant number of patients, in all cases due to being lower that lower limit of quantification. We mitigated this by performing analysis in both versions – with and without extrapolated values and deemed as significant only those discoveries significant in both versions of analysis. Due to large set of studied sICs, there were many hypotheses tested in this discovery study, which may render significant findings questionable due to multiple comparison issue. We did not correct p values for multiple comparison to prevent lowering the sensitivity for potential findings, therefore significant findings are deemed nominal. Due to this, further studies in a larger number of patients and with a smaller subset of studied analytes are needed to confirm these exploratory findings.

To conclude, presented exploratory study discovered association of elevated levels of sTIM-3, sHVEM, sCD27 and sCD40 in the presence of LVSI and in higher stage and risk group tumors, unveiling their potential as prognostic biomarkers in endometrial cancer, measurable in peripheral venous blood. Specifically, these biomarkers may be used as surrogates for EC lymphovascular space invasion in the future, predicting it without obtaining hysterectomy specimen, a fact especially important in younger patients seeking fertility preserving treatment. Additionally, association of elevated levels of sPD-1, sPD-L1, sLAG-3, sICOS, sGITR with mismatch repair deficient endometrial cancer was discovered. Importantly, in addition to mismatch repair deficiency being an adverse prognostic factor, it is also an established predictive biomarker for response to therapy with immune checkpoint inhibitors. Established association may lead to these soluble immune checkpoints be used as surrogate immunotherapy response biomarkers measurable from peripheral blood, avoiding limitations due to spatial and temporal heterogeneity of MMR and other tissue based biomarkers. However, these findings should ideally be confirmed in larger, preferably multicentric validation studies before further clinical development. In contrast to findings from studies in other malignancies and two limited investigations in endometrial cancer, the present study did not identify association of soluble immune checkpoint levels with diagnosis of endometrial cancer. This discrepancy points out to the need for further studies before determining soluble immune checkpoints potential as diagnostic peripheral blood-based biomarkers in endometrial cancer.

## Data Availability

The raw data supporting the conclusions of this article will be made available by the authors, without undue reservation.
