# Supplementary material for: Soluble immune checkpoints in endometrial cancer – a discovery study
Source: Front Immunol. 2025 Nov 21;16:1721822. doi: 10.3389/fimmu.2025.1721822 (PMC12678237; doi:10.3389/fimmu.2025.1721822)
Supplement: Supplementary file 1 [file DataSheet1.pdf]

Soluble immune checkpoints in endometrial cancer – a discovery study

Boštjan Pirš, MD[1,2], Maja Pušić Novak, PhD[2,4], Luka Roškar, MD PhD[2,3], Tea Lanišnik Rižner, PhD[2,4,*], Špela Smrkolj, MD PhD[1,2,*]

[1] Department of Gynaecology, Division of Gynaecology and Obstetrics University Medical Centre Ljubljana, Zaloška 7, 1000 Ljubljana, Slovenia

[2] Faculty of Medicine, University of Ljubljana, Vrazov trg 2, 1000 Ljubljana, Slovenia.

[3] Department of Gynaecology and Obstetrics, General hospital Murska Sobota, Ulica dr. Vrbnjaka 6, 9000 Murska Sobota

[4] Laboratory for Translational Molecular Endocrinology, Institute of Biochemistry and Molecular Genetics, Faculty of Medicine, University of Ljubljana, Vrazov trg 2, 1000 Ljubljana, Slovenia.

*** Correspondence:**

Tea Lanišnik Rižner (tea.lanisnik.rizner@mf.uni-lj.si) and Špela Smrkolj (spela.smrkolj@mf.uni-lj.si) are co-corresponding authors for this manuscript.

Supplementary Table 1. Comorbidities and current medications in study subjects, expressed as absolute (n) and relative frequencies (%). Data for 3 EC patients missing. No differences in comorbidities and current medication use between EC and control patients were observed (Chi square test).

Comorbidities and current medication use				
	All patients (N=73)	EC patients (N=47)	Control patients (N=26)	EC vs. control patients, p-value
Comorbidities				
Diabetes	9 (12.3%)	7 (14.9%)	2 (7.7%)	0.370
Hypertension	36 (49.3%)	24 (51.1%)	12 (46.2%)	0.688
Hyperlipidemia	20 (27.4%)	14 (29.8%)	6 (23.1%)	0.538
Hypothyroidism	3 (4.1%)	3 (6.4%)	0 (0%)	0.188
Asthma or COPD	3 (4.1%)	3 (6.4%)	0 (0%)	0.188
Other autoimmune diseases	0 (0%)	0 (0%)	0 (0%)	
Current medication use				
Aromatase inhibitors	0 (0%)	0 (0%)	0 (0%)	
Tamoxifen	0 (0%	0 (0%)	0 (0%)	
Hormone replacement therapy	0 (0%)	0 (0%)	0 (0%	
Oral contraceptives	0 (0%)	0 (0%)	0 (0%	
Hormonal IUD	0 (0%)	0 (0%)	0 (0%	
Thyroid hormone replacement therapy	3 (4.1%)	3 (6.4%)	0 (0%)	0.188
Antihyperlipemics	19 (26%)	13 (27.7%)	6 (23.1%)	0.669
Inhalatory corticosteroids	3 (4.1%)	3 (6.4%)	0 (0%)	0.188
Antiglycemics	7 (9.6%)	6 (12.8%)	1 (3.8%)	0.215
Coumarins	1 (1.4%)	1 (2.1%)	0 (0%)	0.454
Heparin and LMWH	0 (0%)	0 (0%)	0 (0%)	
NOACS	10 (13.7%)	5 (10.6%)	5 (19.2%)	0.307
Antithrombocyte drugs	73 (100%)	47 (100%)	26 (100%)	
Diuretics	9 (12.3%)	7 (14.9%)	2 (7.7%)	0.370
Beta blockers	14 (19.2%)	11 (23.4%)	3 (11.5%)	0.218
ACE inhibitors	19 (26%)	14 (29.8%)	5 (19.2%)	0.325
Angiotensin receptor blockers	6 (8.2%)	5 (10.6%)	1 (3.8%)	0.312
Calcium channel blockers	4 (5.5%)	4 (8.5%)	0 (0%)	0.126
Vasodilators	1 (1.4%)	1 (2.1%)	0 (0%)	0.454
Aldosterone antagonists	0 (0%)	0 (0%)	0 (0%)	

COPD – chronic obstructive pulmonary disease, IUD – intrauterine device, LMWH – low molecular weight heparin, NOAC – new oral anticoagulants, ACE – angiotensin converting enzyme

Supplementary Table 2. sICs levels and coefficients of variation in control patients, sorted by the latter. * - Analysis excluding extrapolated levels

	N	pg/mL median (IQR)	Coefficient of variation
PD-L2	26	16068.8	0.203
CD40	26	948.3	0.208
TIM-3	26	4487.7	0.293
BTLA	25	3564.9	0.381
BTLA*	25	3564.9	0.381
PD-L1	25	639.9	0.393
PD-L1*	25	639.9	0.393
PD-1	25	2812.6	0.395
HVEM	26	2237.8	0.421
CD86/B7-2*	24	829.3	0.460
CD28	25	8888.7	0.472
LAG-3	26	69651.1	0.473
ICOS	26	7530.9	0.480
CD27	26	2868.7	0.496
CD86/B7-2	25	799.6	0.503
CTLA-4*	19	45	0.518
GITRL	26	849.3	0.535
CD80/B7-1	26	903.1	0.617
CD80/B7-1*	26	903.1	0.617
CTLA-4	26	37.2	0.640
GITR	26	591.7	0.663

pg/mL – picograms per mililiter, IQR – interquartile range

Supplementary table 3. sIC levels for all patients, [a] comparison between control and endometrial cancer (EC) patients (Mann-Whitney U test), [b] p-value of sIC coefficients in robust logistic regression model including specific sIC and endometrial cancer risk factors – age, BMI, parity, waist-to-hip ratio, physical activity, menopausal status, smoking status. * - Analysis excluding extrapolated levels

sIC levels differences between control and EC patients								
	All patients		Control patients		EC patients		p value [a]	p-value [b]
	N	median (IQR)	N	median (IQR)	N	median (IQR)		
sPD-1	75	2552.6 (1349.6, 3508.4)	25	2673.4 (1937.8, 3185.1)	50	1677.6 (877.6, 3637.0)	0.124	0.251
sPD-L1	75	541.5 (271.3, 875.8)	25	652.0 (455.4, 804.1)	50	484.9 (224.4, 935.9)	0.342	0.412
sPD-L1*	74	554.4 (279.9 to 881.0)	25	652.0 (455.4 to 804.1)	49	509.5 (237.3 to 943.8)	0.407	0.451
sPD-L2	76	16747.0 (13754.3, 19133.0)	26	16540.7 (14230.8, 18028.6)	50	17320.4 (13265.8, 19531.9)	0.427	0.365
sCTLA-4	74	30.8 (11.8, 47.7)	25	32.5 (23.5, 47.0)	49	29.7 (10.8, 50.9)	0.274	0.440
sCTLA-4*	45	42.1 (34.8 to 62.3)	19	38.7 (29.9 to 49.8)	26	47.7 (36.4 to 67.4)	0.063	0.097
sLAG-3	76	59929.0 (29946.8, 89254.1)	26	67409.6 (51793.5, 86341.6)	50	45360.6 (21521.4, 89558.7)	0.104	0.088
sTIM-3	75	4269.4 (3274.1, 5175.2)	26	4302.6 (3488.2, 4928.3)	49	4109.4 (3178.7, 5246.7)	0.585	0.357
sBTLA	75	2957.7 (1594.7, 4581.6)	25	3466.4 (2529.2, 4443.1)	50	2586.4 (1241.0, 5191.6)	0.169	0.411
sBTLA*	74	3056.9 (1698.6 to 4581.6)	25	3466.4 (2529.2 to 4443.1)	49	2701.2 (1354.9 to 5394.9)	0.207	0.492
sHVEM	75	2053.2 (1639.2, 2590.8)	26	2127.3 (1771.7, 2604.0)	49	1993.3 (1638.1, 2535.8)	0.654	0.706
sICOS	75	6667.5 (2933.2, 10476.3)	26	7386.5 (5744.7, 9413.0)	49	6413.7 (2277.4, 11168.0)	0.456	0.489

sGITR	75	442.0 (164.7, 775.6)	26	534.1 (323.2, 704.7)	49	370.2 (137.2, 780.6)	0.189	0.358
sGITR*	73	454.4 (192.1 to 780.6)	26	534.1 (323.2 to 704.7)	47	397.4 (147.3 to 803.5)	0.287	0.645
sGITRL	75	777.6 (358.1, 1083.7)	26	801.0 (601.1, 976.0)	49	633.2 (232.3, 1092.0)	0.376	0.291
sCD40	75	867.8 (757.9, 1055.4)	26	904.6 (814.0, 1008.2)	49	850.8 (742.1, 1072.5)	0.311	0.279
sCD27	75	2504.2 (1708.3, 3809.7)	26	2637.7 (2006.5, 3500.1)	49	2405.8 (1648.0, 4408.6)	0.876	0.728
sCD28	75	6756.5 (3537.0, 11777.9)	25	8194.1 (6092.0, 12053.1)	50	6590.4 (3342.0, 11695.3)	0.136	0.295
sCD80/B7-1	75	649.1 (392.6, 1185.8)	26	838.7 (452.9, 1220.6)	49	514.1 (244.4, 1141.3)	0.126	0.141
sCD80/B71*	74	664.2 (401.8 to 1187.2)	26	838.7 (452.9 to 1220.6)	48	536.1 (298.1 to 1151.8)	0.157	0.184
sCD86/B7-2	71	750.7 (283.4, 1151.1)	25	786.5 (570.2, 1000.4)	46	613.0 (216.5, 1322.3)	0.539	0.973
sCD86/B7-2*	61	822.3 (515.5 to 1319.0)	24	786.5 (577.0 to 1018.2)	37	911.5 (377.2 to 1385.2)	0.560	0.259

pg/mL – picograms per mililiter, IQR – interquartile range

Supplementary table 4. sIC levels by tumor histopathological characteristics. Lymphovascular space invasion classified as absent or focal and substantial as per ESGO-ESTRO-ESP 2020 guidelines criteria. Non-aggressive tumors defined as endometrioid low grade (grade 1 or 2) and aggressive as all others according to FIGO 2023 staging. For each characteristic, sIC levels of patients in different subgroups were compared with Mann-Whitney U test. * - Analysis excluding extrapolated levels

sIC levels by tumor histopathological characteristics										
	Lymphovascular space invasion					Histology agressivenes				
	Absent of focal		Substantial		p value	Non-aggressive		Aggressive		p value
	N	pg/mL, median (IQR)	N	pg/mL, median (IQR)		N	pg/mL, median (IQR)	N	pg/mL, median (IQR)	
sPD-1	34	1588.3 (783.3, 3336.9)	16	3067.9 (1601.9, 4834.8)	0.058	33	1650.4 (1005.9, 3714.6)	17	1704.8 (827.1, 3271.3)	0.862
sPD-L1	34	413.6 (171.8, 860.2)	16	701.1 (325.5, 1063.9)	0.096	33	509.5 (237.3, 943.8)	17	407.4 (199.0, 912.0)	0.878
sPD-L1*	33	419.7 (186.7 to 865.4)	16	701.1 (325.5 to 1063.9)	0.125	32	519.3 (238.3 to 964.7)	17	407.4 (199.0 to 912.0)	0.737
sPD-L2	34	17041.1 (13736.0, 19616.2)	16	17490.2 (13028.0, 19070.7)	0.755	33	17201.0 (13516.0, 19641.2)	17	17540.6 (13182.4, 19018.6)	0.493
sCTLA-4	33	23.7 (8.5, 46.0)	15	38.7 (11.6, 72.9)	0.142	31	31.4 (11.2, 52.4)	17	28.0 (7.6, 40.8)	0.553
sCTLA-4*	16	47.7 (35.6 to 64.9)	10	53.9 (39.1 to 87.2)	0.399	17	49.4 (40.4 to 64.9)	9	40.8 (35.7 to 75.7)	0.666
sLAG-3	34	36275.9 (17654.4, 89558.7)	16	64269.9 (40483.0, 86908.7)	0.220	33	43855.3 (24931.6, 90598.0)	17	47337.7 (15162.2, 73615.0)	0.675
sTIM-3	34	**3515.3 (3018.2, 4539.7)**	15	**5572.7 (4293.0, 6719.6)**	**< 0.001**	33	4109.4 (3223.4, 5246.7)	16	4143.9 (3112.6, 5222.1)	0.881
sBTLA	34	1992.6 (979.8, 4318.1)	16	3325.3 (2000.4, 6588.6)	0.070	33	2211.6 (1354.9, 5491.0)	17	2701.2 (1203.1, 3606.8)	0.766
sBTLA*	33	2153.6 (1049.6 to 4415.4)	16	3325.3 (2000.4 to 6588.6)	0.092	32	2499.2 (1377.5 to 5580.1)	17	2701.2 (1203.1 to 3606.8)	0.629
sHVEM	34	**1876.5 (1628.3, 2228.1)**	15	**2735.6 (1692.0, 3371.7)**	**0.037**	33	1892.6 (1673.9, 2535.8)	16	2070.6 (1467.9, 2440.1)	0.765

sICOS	33	4265.3 (2277.4, 10612.1)	16	7179.2 (2745.0, 13819.1)	0.228	32	5372.9 (2364.5, 11232.0)	17	7555.9 (2277.4, 10612.1)	0.966
sGITR	33	216.9 (117.1, 709.8)	16	532.9 (230.5, 1079.7)	0.070	32	396.2 (147.3, 920.5)	17	271.3 (91.8, 714.8)	0.383
sGITR*	31	271.3 (134.7 to 712.3)	16	532.9 (230.5 to 1079.7)	0.121	31	422.2 (157.3 to 961.0)	16	334.4 (107.0 to 731.3)	0.445
sGITRL	33	533.2 (164.7, 1077.3)	16	982.6 (515.0, 1199.1)	0.107	32	793.2 (300.1, 1150.4)	17	529.0 (232.3, 984.5)	0.266
sCD40	34	**793.2 (680.4, 995.4)**	15	**1090.1 (798.9, 1299.8)**	**0.017**	33	792.0 (715.5, 1055.9)	16	886.4 (788.6, 1102.4)	0.277
sCD27	34	**2265.0 (1393.3, 3133.8)**	15	**4348.0 (2222.7, 6458.1)**	**0.018**	33	2504.2 (1643.3, 4648.7)	16	2297.9 (1690.5, 3354.6)	0.565
sCD28	34	**4177.1 (3007.4, 11585.2)**	16	**6867.1 (4954.6, 13397.3)**	**0.048**	33	6645.8 (3314.1, 12823.2)	17	6535.1 (3425.6, 7420.3)	0.413
sCD80/B7-1	33	464.3 (136.7, 1141.3)	16	700.5 (429.6, 1225.0)	0.245	32	581.6 (331.6, 1209.4)	17	430.2 (244.4, 809.8)	0.475
sCD80/B7-1*	32	480.1 (168.5 to 1151.8)	16	700.5 (429.6 to 1225.0)	0.309	31	649.1 (410.8 to 1235.7)	17	430.2 (244.4 to 809.8)	0.360
sCD86/B7-2	30	474.2 (180.0, 1314.6)	16	846.9 (302.3, 1931.4)	0.278	30	660.1 (218.9, 1380.8)	16	603.9 (206.7, 1022.6)	0.580
CD86/B7-2*	23	956.0 (358.6 to 1345.5)	14	880.3 (476.9 to 1964.6)	0.443	24	947.1 (388.8 to 1510.9)	13	849.1 (377.2 to 1062.5)	0.382

pg/mL – picograms per mililiter, IQR – interquartile range

Supplementary table 5. sIC levels by tumor molecular characteristicsC Expression of p53 was determined as abberant or wild type by IHC. Mismatch repair status was determined by expression of MLH1, PMS2, MSH2 and MSH6 by IHC – tumors with absent expression in one or more of those proteins were deemed as deficient, otherwise as proficient. For each characteristic, sIC levels of patients in different subgroup were compared with Mann-Whitney U test. * - Analysis excluding extrapolated levels

sIC levels by tumor molecular markers										
	Mismatch repair status					p53 expression				
	Proficient		Deficient		p value	Wild type		Abberant		p value
	N	pg/mL, median (IQR)	N	pg/mL, median (IQR)		N	pg/mL, median (IQR)	N	pg/mL, median (IQR)	
sPD-1	34	1588.3 (678.1, 3283.0)	16	3109.1 (1560.2, 4890.7)	**0.027**	43	1650.4 (801.8, 3970.6)	7	1704.8 (1475.7, 2459.2)	0.878
sPD-L1	34	360.6 (171.8, 815.4)	16	837.2 (364.9, 1369.4)	**0.018**	43	509.5 (228.7, 965.9)	7	407.4 (256.4, 783.2)	0.834
sPD-L1*	33	407.4 (186.7 to 844.5)	16	837.2 (364.9 to 1369.4)	**0.024**	42	519.3 (237.6 to 976.9)	7	407.4 (256.4 to 783.2)	0.753
sPD-L2	34	17320.4 (14132.3, 19405.8)	16	17010.0 (12459.9, 21253.8)	0.983	43	17201.0 (13152.2, 19522.6)	7	18397.0 (15896.7, 19290.3)	0.812
sCTLA-4	32	25.9 (9.5, 39.1)	16	44.0 (11.7, 72.8)	0.066	41	31.4 (11.0, 55.4)	7	28.0 (14.0, 31.8)	0.672
sCTLA-4*	16	39.5 (32.7 to 64.9)	10	61.2 (46.8 to 91.3)	**0.014**	22	52.4 (40.5 to 67.4)	4	31.8 (28.0 to 45.7)	0.102
sLAG-3	34	38928.0 (15620.0, 78232.4)	16	73426.2 (35825.2, 98619.3)	**0.038**	43	46866.0 (22473.6, 90154.5)	7	43593.7 (24481.9, 54761.2)	0.567
sTIM-3	34	4082.6 (3248.7, 4994.5)	15	4829.6 (3162.3, 5773.0)	0.461	42	4286.4 (3248.7, 5265.0)	7	3417.7 (3100.0, 4024.5)	0.130
sBTLA	34	2022.0 (979.8, 4318.1)	16	3409.7 (1881.5, 6714.6)	0.088	43	2211.6 (1057.3, 5449.8)	7	2701.2 (2151.7, 3345.3)	0.911
sBTLA*	33	2153.6 (1049.6 to 4415.4)	16	3409.7 (1881.5 to 6714.6)	0.115	42	2499.2 (1137.5 to 5470.4)	7	2701.2 (2151.7 to 3345.3)	0.989
sHVEM	34	1943.0 (1629.7, 2298.0)	15	1996.8 (1666.4, 2802.4)	0.544	42	1943.0 (1647.0, 2685.7)	7	2053.9 (1560.2, 2274.1)	0.732

sICOS	33	4140.5 (1840.5, 9044.9)	16	9179.5 (4234.1, 14495.5)	**0.040**	42	5372.9 (1987.9, 11359.9)	7	8040.4 (5785.6, 9828.5)	0.361
sGITR	33	266.4 (112.1, 599.1)	16	645.7 (210.7, 972.0)	**0.046**	42	396.2 (139.8, 866.6)	7	271.3 (164.5, 498.2)	0.568
sGITR*	32	268.8 (115.9 to 628.0)	15	709.8 (224.3 to 1064.1)	**0.020**	40	453.2 (147.3 to 920.5)	7	271.3 (164.5 to 498.2)	0.420
sGITRL	33	533.2 (164.7, 1033.0)	16	1002.2 (510.5, 1659.2)	0.050	42	793.2 (211.9, 1135.8)	7	529.0 (273.9, 679.7)	0.530
sCD40	34	813.6 (680.4, 1057.3)	15	985.7 (759.2, 1211.7)	0.165	42	851.5 (722.2, 1088.7)	7	832.9 (782.8, 968.7)	0.977
sCD27	34	2297.9 (1652.7, 3347.3)	15	2852.9 (1614.5, 4955.3)	0.329	42	2582.6 (1652.7, 4723.9)	7	2199.3 (1486.6, 2466.6)	0.162
sCD28	34	5759.3 (3105.0, 9738.5)	16	9462.2 (4038.0, 12617.0)	0.081	43	6645.8 (3369.9, 12135.6)	7	3871.1 (3314.1, 7033.1)	0.371
sCD80/B7-1	33	464.3 (136.7, 870.9)	16	791.5 (441.0, 1619.4)	0.054	42	505.0 (149.1, 1172.6)	7	612.0 (373.1, 725.5)	0.886
sCD80/B7-1*	32	480.1 (138.5 to 928.6)	16	791.5 (441.0 to 1619.4)	0.071	41	514.1 (179.1 to 1183.0)	7	612.0 (373.1 to 725.5)	0.804
sCD86/B7-2	31	432.9 (128.1, 982.6)	15	1062.5 (372.5, 1835.4)	**0.019**	39	692.2 (201.9, 1376.4)	7	515.5 (296.9, 982.6)	0.783
sCD86/B7-2*	22	846.9 (391.1 to 1317.9)	15	1062.5 (372.5 to 1835.4)	0.315	31	911.5 (372.5 to 1570.5)	6	735.7 (411.8 to 996.0)	0.376

pg/mL – picograms per mililiter, IQR – interquartile range

Supplementary table 6. sIC levels by anatomical stage - differences in sIC levels between patients with uterus confined disease (FIGO 2023 stage IIC or lower) and patients with locally or regionally advanced or metastatic disease (FIGO 2023 stage IIIA or higher). Comparison with Mann-Whitney U test. * - Analysis excluding extrapolated levels

sIC levels by anatomical stage					
	Uterus confined		Advanced/metastatic		p value
	N	pg/mL, median (IQR)	N	pg/mL, median (IQR)	
sPD-1	42	1677.6 (877.6, 3541.8)	8	2398.7 (1143.6, 4469.8)	0.701
sPD-L1	42	484.9 (204.3, 905.5)	8	536.3 (255.8, 1018.0)	0.711
sPD-L1*	41	509.5 (220.0 to 912.0)	8	536.3 (255.8 to 1018.0)	0.787
sPD-L2	42	16731.8 (13220.4, 19433.7)	8	18656.9 (16451.0, 20372.9)	0.354
sCTLA-4	40	29.7 (11.0, 46.8)	8	24.8 (9.5, 67.1)	0.967
sCTLA-4*	22	44.0 (35.4 to 64.9)	4	67.3 (59.9 to 70.3)	0.286
sLAG-3	42	43724.5 (19871.1, 90376.2)	8	54380.0 (24793.2, 85588.3)	0.895
sTIM-3	42	3725.2 (3154.2, 5048.6)	7	5213.3 (4747.1, 6176.7)	0.056
sBTLA	42	2586.4 (1241.0, 5191.6)	8	2596.6 (1617.8, 3993.6)	1
sBTLA*	41	2701.2 (1354.9 to 5394.9)	8	2596.6 (1617.8 to 3993.6)	0.914
sHVEM	42	1886.8 (1629.2, 2298.0)	7	3220.5 (1925.9, 3628.3)	0.072
sICOS	41	6413.7 (2541.9, 11168.0)	8	5326.9 (1713.6, 11424.6)	0.745

sGITR	41	370.2 (137.2, 714.8)	8	463.9 (133.4, 882.9)	0.968
sGITR*	39	397.4 (157.3 to 742.6)	8	463.9 (133.4 to 882.9)	0.854
sGITRL	41	633.2 (242.3, 1092.0)	8	682.6 (200.5, 1052.9)	0.675
sCD40	42	813.6 (722.2, 1049.1)	7	1296.1 (1029.9, 1347.7)	**0.013**
sCD27	42	2265.0 (1469.1, 3381.8)	7	4348.0 (2493.6, 5172.2)	**0.040**
sCD28	42	6424.3 (3314.1, 11695.3)	8	6645.8 (4843.2, 8273.2)	0.791
sCD80/B7-1	41	514.1 (244.4, 1183.0)	8	498.7 (288.9, 1111.6)	0.935
sCD80/B7-1*	40	563.1 (298.1 to 1194.0)	8	498.7 (288.9 to 1111.6)	0.846
sCD86/B7-2	38	613.0 (218.9, 1322.3)	8	561.5 (206.7, 1138.8)	0.739
sCD86/B7-2*	30	933.7 (412.0 to 1434.8)	7	849.1 (245.2 to 1215.0)	0.362

pg/mL – picograms per mililiter, IQR – interquartile range
